# Long-term findings from COMFORT-II, a phase 3 study of ruxolitinib vs best available therapy for myelofibrosis

**DOI:** 10.1038/leu.2016.148

**Published:** 2016-06-17

**Authors:** C N Harrison, A M Vannucchi, J-J Kiladjian, H K Al-Ali, H Gisslinger, L Knoops, F Cervantes, M M Jones, K Sun, M McQuitty, V Stalbovskaya, P Gopalakrishna, T Barbui

**Affiliations:** 1 Guy's and St Thomas' NHS Foundation Trust, Guy's Hospital, London, UK; 2 Center for Research and Innovation for Myeloproliferative Neoplasms-CRIMM, AOU Careggi, Department of Experimental and Clinical Medicine, University of Florence, Florence, Italy; 3 Hôpital Saint-Louis et Université Paris Diderot, Paris, France; 4 University of Leipzig, Leipzig, Germany; 5 Medical University of Vienna, Vienna, Austria; 6 Cliniques Universitaires Saint-Luc and de Duve Institute, Université catholique de Louvain, Brussels, Belgium; 7 Hospital Clínic, Institut d'Investigacions Biomèdiques August Pi i Sunyer, Barcelona, Spain; 8 Incyte Corporation, Wilmington, DE, USA; 9 Novartis Pharma, Basel, Switzerland; 10 Research Foundation, Ospedale Papa Giovanni XXIII, Bergamo, Italy

## Abstract

Ruxolitinib is a Janus kinase (JAK) (JAK1/JAK2) inhibitor that has demonstrated superiority over placebo and best available therapy (BAT) in the Controlled Myelofibrosis Study with Oral JAK Inhibitor Treatment (COMFORT) studies. COMFORT-II was a randomized (2:1), open-label phase 3 study in patients with myelofibrosis; patients randomized to BAT could crossover to ruxolitinib upon protocol-defined disease progression or after the primary end point, confounding long-term comparisons. At week 48, 28% (41/146) of patients randomized to ruxolitinib achieved ⩾35% decrease in spleen volume (primary end point) compared with no patients on BAT (*P*<0.001). Among the 78 patients (53.4%) in the ruxolitinib arm who achieved ⩾35% reductions in spleen volume at any time, the probability of maintaining response was 0.48 (95% confidence interval (CI), 0.35–0.60) at 5 years (median, 3.2 years). Median overall survival was not reached in the ruxolitinib arm and was 4.1 years in the BAT arm. There was a 33% reduction in risk of death with ruxolitinib compared with BAT by intent-to-treat analysis (hazard ratio (HR)=0.67; 95% CI, 0.44–1.02; *P*=0.06); the crossover-corrected HR was 0.44 (95% CI, 0.18–1.04; *P*=0.06). There was no unexpected increased incidence of adverse events with longer exposure. This final analysis showed that spleen volume reductions with ruxolitinib were maintained with continued therapy and may be associated with survival benefits.

## Introduction

Myelofibrosis (MF) is a myeloproliferative neoplasm primarily characterized by fibrosis of the bone marrow, cytopenias, extramedullary hematopoiesis and dysregulation of the Janus kinase (JAK)/signal transducer and activator of transcription pathway.^1, 2^ Patients often have varying degrees of splenomegaly as well as constitutional symptoms (that is, fever, weight loss and night sweats) and symptoms related to the disease (for example, fatigue, pruritus and abdominal pain), all of which have a significant impact on the quality of life.^3, 4^ Survival in MF is also reduced, especially for patients with high- and intermediate-2-risk disease, who have a median life expectancy of approximately 1.5–4 years.^5, 6^

Ruxolitinib is a potent and selective JAK1/JAK2 inhibitor^7^ that has demonstrated superiority over best available therapy (BAT) in the phase 3 Controlled Myelofibrosis Study with Oral JAK Inhibitor Treatment II (COMFORT-II) trial.^8^ Patients receiving ruxolitinib experienced rapid and durable reductions in splenomegaly, improved MF-related symptoms and quality of life measures, and prolonged overall survival (OS) compared with patients receiving BAT. In addition, several analyses have reinforced the apparent survival benefit with ruxolitinib compared with placebo (in COMFORT-I)^9, 10^ or historical cohorts of patients not treated with JAK inhibitors.^11, 12^ On the basis of the findings from the COMFORT studies, ruxolitinib has been approved for the treatment of MF.

Long-term comparisons in the COMFORT trials are limited by the crossover design, in which patients were allowed to crossover from the control arms to receive ruxolitinib after a relatively short period of time on comparator treatment (6 months in COMFORT-I and 12 months in COMFORT-II). At later follow-up times, these analyses compared patients who had been receiving ruxolitinib since randomization with those who had been receiving ruxolitinib after a delayed start. Findings from COMFORT-I illustrated this confounding effect, with an apparent diminishing impact of ruxolitinib over placebo the later the intent-to-treat (ITT) analysis of OS was conducted after crossover. Survival at the 1-year (hazard ratio (HR), 0.50; *P*=0.04)^9^ and 2-year analyses (HR, 0.58; *P*=0.03)^10^ significantly favored ruxolitinib vs placebo; however, the difference was no longer statistically significant at the time of the 3-year analysis (HR, 0.69; *P*=0.067).^13^ Interestingly, the OS benefit at the 3-year follow-up of COMFORT-II was statistically significant in an ITT analysis despite all remaining BAT patients having already crossed over to ruxolitinib and having received treatment for a number of years;^14^ this is likely due to the later crossover time allowed per protocol vs COMFORT-I.

Here in this 5-year final analysis, we report long-term safety and efficacy results in patients treated with ruxolitinib in the COMFORT-II trial. Because the crossover design of the trial limits comparison of OS in an ITT analysis, we conducted statistical modeling using rank-preserving structural failure time (RPSFT) analysis^15, 16^ to approximate the full impact of ruxolitinib on survival compared with BAT. The RPSFT method is recognized by health authorities as a way to correct for crossover between treatment arms.^17^

## Materials and methods

### Study design

COMFORT-II was a multicenter, open-label, phase 3 study comparing the safety and efficacy of ruxolitinib with that of BAT in MF. The trial population included patients diagnosed with primary MF, post-polycythemia vera MF (PPV-MF) or post-essential thrombocythemia MF (PET-MF) per 2008 World Health Organization criteria^18^ and classified as International Prognostic Scoring System (IPSS) intermediate-2 or high risk.^5^ Patients were stratified by IPSS risk groups and randomized 2:1 to receive starting doses of ruxolitinib 15 or 20 mg twice daily (bid; based on baseline platelet counts of 100–200 × 10^9^/l or >200 × 10^9^/l, respectively) or investigator-determined BAT. Ruxolitinib could be titrated over the course of treatment, to a maximum of 25 mg bid, to optimize safety and efficacy for each patient; BAT included any commercially available therapy (single agent or combination) or observation only and could be changed during the treatment phase. Patients were allowed to crossover from the BAT arm to receive ruxolitinib upon protocol-defined progressive splenomegaly, defined as a ⩾25% increase in spleen volume from on-study nadir. The full details of the study design and patient enrollment criteria have been described previously.^8^ This final report is based on the database lock for the study, performed on 20 April 2015.

The study was approved by the institutional review boards of the respective institutions before patient enrollment and was conducted in accordance with the principles of the Declaration of Helsinki. All patients provided written informed consent. The trial is registered with ClinicalTrials.gov (NCT00934544).

### Efficacy analyses

The primary efficacy end point was the proportion of patients achieving a ⩾35% reduction from baseline in spleen volume, as measured by magnetic resonance imaging (that is, a spleen response), at week 48 and was compared using the Fisher's exact test. Additional efficacy end points included the duration of spleen response, OS and bone marrow histomorphology; change in *JAK2* V617F allele burden was an exploratory end point in the trial. Duration of spleen response was analyzed by the Kaplan–Meier (K-M) method, with loss of response defined as an increase in spleen volume by >25% above the on-study nadir that was no longer a ⩾35% reduction from baseline. Allele burden by visit was summarized only for patients with positive *JAK2* V617F values at baseline and was measured in blood samples using allele-specific real-time quantitative PCR (RQ-PCR) as described by Levine *et al.*^19^ using an Applied Biosystems ABI 7900 real-time PCR analyzer (Foster City, CA, USA). Patients were categorized relative to an absolute decrease of 20 percentage points. Bone marrow fibrosis was graded according to the European consensus criteria^20^ by an experienced hematopathologist at each study site; the last available postbaseline fibrosis assessment was used for the evaluation of on-treatment changes in fibrosis grade. Patient-reported outcomes were not assessed beyond week 48 or for patients who entered the extension phase.

OS was estimated using K-M analysis, and the event was death from any cause since randomization. The HR and the corresponding 95% CI were estimated using the Cox proportional hazards model stratified by baseline IPSS risk category. The *P*-value was determined using a stratified two-sided log-rank test and is intended for descriptive purposes only. An RPSFT model^15, 16^ was used to estimate the crossover-corrected treatment effect. Using RPSFT analysis maintains the original randomized group definitions, preserving the validity of comparisons between groups and providing a randomization-based estimate of the treatment effect corrected for bias from crossover between groups. The main assumption is that treatment affects OS by multiplying the survival time by a given factor once the patient crosses over and starts receiving ruxolitinib (a structural and untestable assumption). OS was determined for (1) the observed time for patients randomized to ruxolitinib, (2) the observed time for patients randomized to BAT and (3) the observed time for patients randomized to BAT who did not cross over and the reconstructed survival time for those who did crossover to ruxolitinib.

### Safety

Safety results are summarized for the randomized and extension phases for patients randomized to ruxolitinib and separately for all patients who received ruxolitinib at any time on study (as randomized treatment, in the extension phase, or after crossover from BAT). For patients randomized to BAT, safety results are summarized for the duration of randomized treatment until crossover and separately after crossover to ruxolitinib treatment in the extension phase.

## Results

### Patients

Of the 219 patients evaluated during the course of the study, 146 patients were randomized to ruxolitinib and 73 patients were randomized to BAT. After the primary analysis at week 48, all patients remaining on study entered into the extension phase, including 45 patients initially randomized to BAT who crossed over to receive ruxolitinib (median time to crossover by K-M estimate, 75 weeks). At study completion, 39 patients (26.7%) in the ruxolitinib arm and 11 of the 45 patients (24.4%) who crossed over from the BAT arm to receive ruxolitinib completed 5 years of on-study treatment (Table 1; Supplementary Figure 1); these patients were still receiving treatment benefit and were offered commercially available ruxolitinib or enrollment in the compassionate use program following completion of the trial. Primary reasons for premature discontinuation before 5 years were adverse events (AEs; 24.0%) and disease progression (21.9%) in the ruxolitinib arm and withdrawal of consent (12.3%) and other in the BAT arm (12.3% including investigator decision (*n*=3) and stem cell transplant (*n*=2)). The median duration of follow-up was 4.3 years from randomization to the last contact date (ruxolitinib, 4.7 years (range, 0.08–5.5 years); BAT, 2.9 years (range, 0.04–5.41 years)).

Patient demographic and baseline clinical characteristics were well balanced between treatment arms and have been previously described, along with results from the primary analysis.^8^ Disease and hematologic characteristics were representative of a population of patients with advanced primary or secondary MF. Overall, 53% of patients were diagnosed with primary MF (PPV-MF, 31% PET-MF, 16%) and the median age was 65.2 years (range, 35–85 years). Approximately 60 and 40% of patients in both arms were confirmed as having IPSS intermediate-2- or high-risk MF, respectively.

### Efficacy

**Figure 1:**
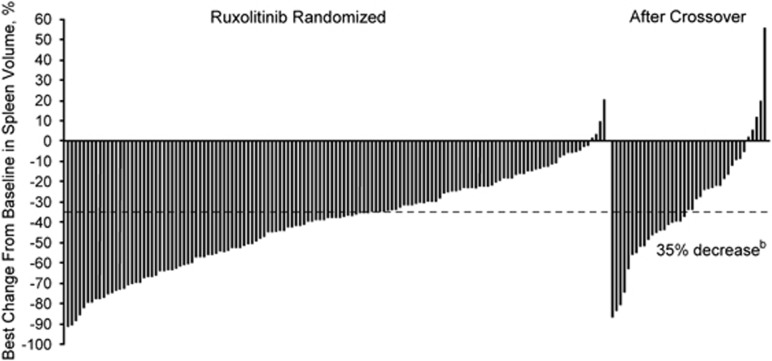
Best change from baseline in spleen volume for individual patients.^a a^Only patients with baseline and ⩾1 postbaseline spleen volume assessments were included (ruxolitinib, *n*=136; BAT crossover, *n*=39). ^b^Patients with a ⩾35% reduction in spleen volume were considered as responders.

**Figure 2:**
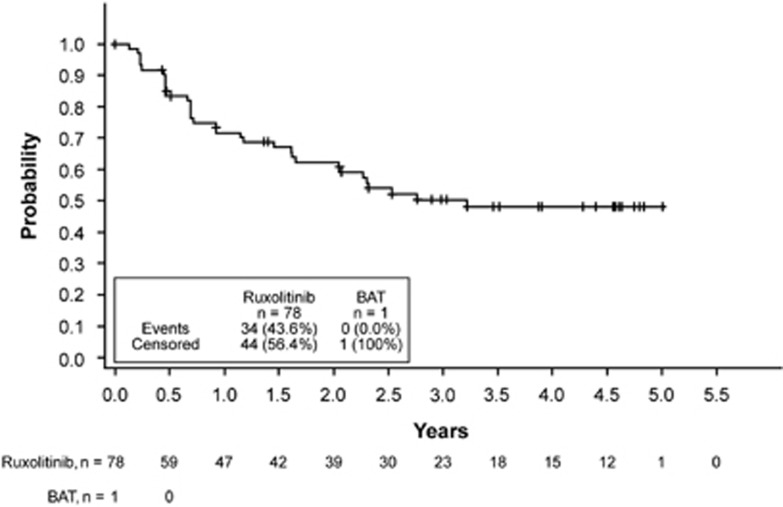
Duration of maintenance of spleen response.^a a^Defined as the interval from first spleen volume measurement of ⩾35% reduction from baseline at any time on study and the first scan that is no longer a 35% reduction *and* that is a >25% increase over on-study nadir.

As reported previously, 28% of patients randomized to ruxolitinib achieved the primary end point compared with no patients who received BAT (*P*<0.001).^8^ Overall, 78 patients (53.4%) in the ruxolitinib arm achieved a ⩾35% reduction in spleen volume at any time on treatment and 97.1% of patients (132/136) with postbaseline spleen assessments experienced a clinical benefit with some degree of spleen volume reduction (Figure 1). In addition, 75.6% of patients (34/45) who crossed over to ruxolitinib treatment from the BAT arm experienced reduction in spleen volume from their last precrossover assessment and 42.2% (19/45) had a ⩾35% reduction at any time after initiation of ruxolitinib therapy. Spleen reductions of ⩾35% among ruxolitinib-randomized patients were sustained with long-term therapy (median duration, 3.2 years; Figure 2); the probability of maintaining a spleen response was 0.51 (95% CI, 0.38–0.62) at 3 years and 0.48 (95% CI, 0.35–0.60) at 5.0 years for patients initially randomized to ruxolitinib.

**Figure 3:**
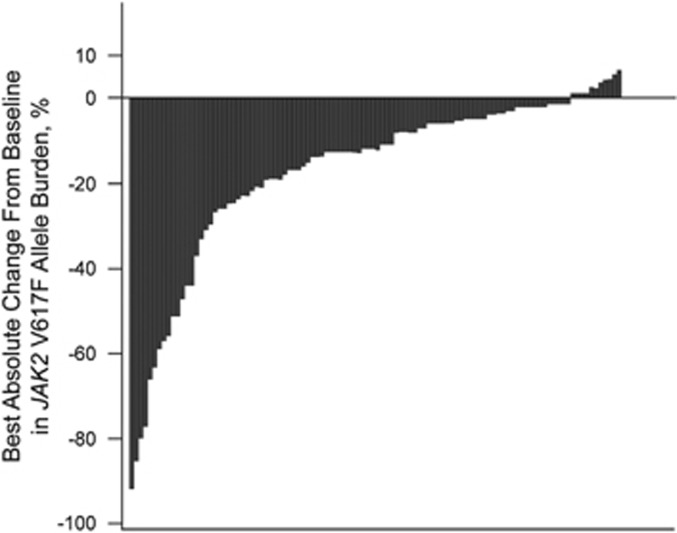
Best absolute reduction in *JAK2* V617F allele burden for individual patients.^a a^Only ruxolitinib-randomized patients with positive Janus kinase 2 (*JAK2*) V617F mutation status at baseline and ⩾1 postbaseline assessment were included (*n*=108).

At baseline, 110 patients were *JAK2* V617F positive, with a median allele burden of 84%, and most of these patients (*n*=107) had an allele burden of ⩾ 20%. Over the course of treatment, approximately one-third of evaluable *JAK2* V617F-positive patients had a >20% reduction from baseline in absolute allele burden at week 168 (38.3% (18/47)) and week 192 (31.0% (13/42)). Among evaluable patients, 74.5 and 83.3% had no increase in allele burden at weeks 168 and 192, respectively. The majority of patients had a reduction in allele burden over the course of ruxolitinib treatment (Figure 3).

Among patients randomized to ruxolitinib, 23 (15.8%) had improved fibrosis (including 4 who improved to grade 0 from baseline fibrosis grades of 1 (*n*=1), 2 (*n*=2) and 3 (*n*=1)), 47 (32.2%) had stable fibrosis and 27 (18.5%) had a worsening of fibrosis by 1 grade (*n*=21) or 2 grades (*n*=6) at their last assessment (median treatment duration, 2.2 years in the ruxolitinib arm and <1 year in the BAT arm). Among patients randomized to BAT, 2 (2.7%) had improved fibrosis, 13 (17.8%) were stable and 4 (5.5%) had worsened fibrosis at their last assessment during randomized treatment (see Supplementary Table 1); however, the majority of patients in the BAT arm had missing baseline or postbaseline assessments, precluding meaningful comparisons between treatment arms.

### Survival

**Figure 4:**
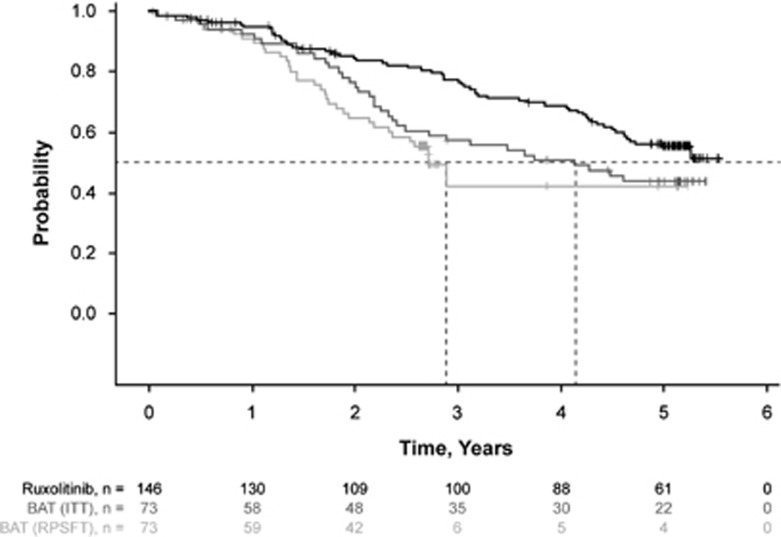
Kaplan–Meier analysis of OS by ITT analysis and RPSFT corrected for crossover from the BAT arm.

Overall, 59 (40.4%) and 35 (47.9%) deaths were reported in the ruxolitinib and BAT arms, respectively. Median OS was not reached in the ruxolitinib arm and was 4.1 years in the BAT arm. In the ITT analysis, patients randomized to ruxolitinib had longer OS compared with those randomized to BAT, with a 33% reduction in risk of death with ruxolitinib treatment (HR, 0.67 (95% CI, 0.44–1.02); *P*=0.06); the K-M estimated probability of survival at 5 years was 56% with ruxolitinib and 44% with BAT (Figure 4). However, the confounding effect on OS of crossover from BAT to ruxolitinib became apparent in this extended follow-up compared with previous analyses.^14, 21^ After adjustment was recensored in the RPSFT model, the number of deaths in the BAT group was 32, with a median survival of 2.7 years, and the crossover-corrected HR for OS was 0.44 (95% CI, 0.18–1.04) in favor of ruxolitinib vs BAT (Figure 4).

### Safety

The median duration of exposure to ruxolitinib was 2.6 years (range, 0.02–5.3 years) in the ruxolitinib arm and 1.2 years (range, 0.04–4.3 years) in the BAT arm after crossover to ruxolitinib. For patients who initiated ruxolitinib at 20 mg bid (*n*=90), the median dose intensity remained stable to week 48 (39.7 mg/day) and then decreased slightly over time (34.6 mg/day at week 96); for patients who started on 15 mg bid (*n*=56), the median dose intensity decreased over the first 24 weeks of therapy (21.6 mg/day at week 24), after which it stabilized at approximately 20 mg/day (21.4 mg/day at week 96). Safety findings should be considered within the context of different exposure durations in the ruxolitinib and BAT arms. Patients randomized to BAT had a median duration of exposure of 0.87 years (45 weeks) because BAT treatment would end with crossover to ruxolitinib as allowed per protocol.

There was no relevant increase in the incidence of AEs with longer exposure compared with previous reports, and there were no new or unexpected AEs.^8, 14^ The most commonly reported non-hematologic AEs in patients who received ruxolitinib at any time (during randomized treatment, in the extension phase or after crossover from BAT) were diarrhea (35.6%) and peripheral edema (33.0%). After adjustment for exposure to study drug (Table 2), the rates of non-hematologic AEs were generally lower with longer-term ruxolitinib treatment and when compared with those in the BAT arm. Infections of special interest among ruxolitinib-treated patients included urinary tract infection (24.6%), pneumonia (13.1%), herpes zoster infection (11.5%), sepsis and septic shock (7.9%), and tuberculosis (1.0%). Incidences of thromboembolic events during the study were negligible and similar between the ruxolitinib and BAT arms (portal vein thrombosis, 1.0 vs 1.4% aortic thrombosis, 0 vs 1.4% and venous thrombosis/deep vein thrombosis, 1.0 vs 1.4%).

Consistent with previous reports and the mechanism of action of ruxolitinib as a potent JAK2 inhibitor, median hemoglobin levels in the ruxolitinib arm decreased over the first 12 weeks of treatment and then recovered to greater than the 100-g/l threshold considered to be the criterion for anemia. Median hemoglobin at week 192 in the ruxolitinib arm was 110.5 g/l compared with 106.0 g/l at baseline. Among all patients who received ruxolitinib (including after crossover from BAT), new or worsening grade 3/4 hemoglobin and lymphocyte decreases were evident (hemoglobin decrease, 46.1% (88/191); lymphocyte decrease, 31.4% (60/191)); decreases to grade 3/4 severity in other hematology parameters occurred in <20% of patients treated with ruxolitinib (thrombocytopenia, 18.8; neutropenia, 8.9; and leukopenia, 6.3%).

Common grade 3/4 AEs included anemia (22.5%), thrombocytopenia (15.2%), pneumonia (5.8%), general physical health deterioration (4.2%) and dyspnea (4.2%); exposure-adjusted rates of grade 3/4 AEs are shown in Table 3. Approximately one-half of the enrolled patients (55.0% overall, 58.2% of patients in the ruxolitinib plus extension phases and 44% of patients who crossed over from BAT to ruxolitinib) experienced ⩾1 serious AE (SAE). SAEs included anemia (6.3%), pneumonia (6.3%), abdominal pain (3.7%), thrombocytopenia (3.1%) and cardiac failure (3.1%). SAEs of or related to bleeding were infrequent and occurred in <2% of patients who received ruxolitinib at any time.

Eight patients (5.5%) in the ruxolitinib arm and five patients (6.8%) in the BAT arm developed leukemia. Overall, 25 patients (17.1%) in the ruxolitinib arm and 2 patients (2.7%) in the BAT arm had newly diagnosed non-melanoma skin cancer (basal cell or squamous cell carcinoma); after adjustment by patient exposure, rates were 6.1 and 3.0/100 patient-years for ruxolitinib and BAT, respectively.

AEs leading to study drug discontinuation were reported in approximately 25% of patients (24.7% of patients in the ruxolitinib plus extension phases and 26.7% of patients who crossed over from BAT to ruxolitinib). These included thrombocytopenia (*n*=7 (3.7%)) and anemia, splenomegaly, pneumonia and prostate cancer (*n*=2 for each (1.0% each)) occurring in >1 patient; all other AEs that led to discontinuation occurred in 1 patient each. SAEs occurring after discontinuation in >1 patient who received ruxolitinib either in the ruxolitinib arm or after crossover from BAT included acute renal failure (*n*=3), cardiac failure (*n*=3), cerebral hemorrhage (*n*=2), pneumonia (*n*=2), supraventricular tachycardia (*n*=2) and thrombocytopenia (*n*=2). The two patients with cerebral hemorrhage had platelet counts of 116 and 43 × 10^9^/l at the time of the events, and neither were suspected to be related to treatment.

Among all patients who received ruxolitinib, 21 deaths (11%) occurred on or within 28 days of discontinuing treatment (17 in the ruxolitinib randomized plus extension phase and 4 in the ruxolitinib after crossover phase). Causes of death are listed in Supplementary Table 2, none of which were related to the discontinuation of ruxolitinib treatment.

## Discussion

The primary analysis of the COMFORT-II study showed that ruxolitinib treatment resulted in rapid and durable benefits to patients in terms of spleen volume reductions, symptom relief and improvements in quality of life measures.^8^ These 5-year findings show that the initial improvements in splenomegaly were maintained with continuous long-term therapy. Upon crossing over to ruxolitinib, patients randomized to BAT also achieved substantial spleen size reductions, similar to what was observed in the ruxolitinib arm. A survival advantage was apparent in patients randomized to ruxolitinib compared with patients randomized to BAT (median not reached vs median of 4.1 years with BAT), despite the majority of BAT patients crossing over to receive ruxolitinib during the course of the study. This observation indicates a plausible clinical and survival advantage with earlier treatment. In addition to this, and consistent with other reports,^22, 23^ some patients experienced reductions in *JAK2* V617F allele burden, and a notable proportion of patients (48%) maintained or had improvement in their bone marrow fibrosis with longer-term ruxolitinib use. This is also supported by an analysis by Kvasnicka *et al.*,^24^ which showed that patients who received ruxolitinib in the phase 1/2 study experienced stabilization or improvement in bone marrow fibrosis, whereas those in a historical comparator who received standard therapy worsened over time. Taken together, these data suggest greater overall benefits with earlier and/or prolonged ruxolitinib treatment. However, the prognostic implications of improvements, or worsening, in bone marrow fibrosis or *JAK2* V617F allele burden are unclear; given the prognostic significance of spleen size reduction with ruxolitinib treatment^25^ and the benefit that symptom improvement provides to patients, monitoring response to treatment in clinical practice by evaluating spleen size and symptoms, rather than with fibrotic score or allele burden, provides the best assessment of treatment benefit.

The long-term safety and tolerability of ruxolitinib were consistent with previous findings. As expected, given the mechanism of action of ruxolitinib as a JAK1/JAK2 inhibitor, the most common hematologic AEs were anemia and thrombocytopenia, but these rarely led to treatment discontinuation. Thrombocytopenia was managed effectively with dose modifications, with rare instances of permanent discontinuations of therapy. Anemia was generally manageable with supportive care, and the predictable decrease in hemoglobin levels that occurred during the first 12 weeks of ruxolitinib therapy returned to near baseline levels after approximately 24 weeks of treatment.^13, 14^ Moreover, the hemoglobin changes that occurred with ruxolitinib treatment did not bear the same prognostic implications as those that occur as a consequence of MF pathology. An analysis of hemoglobin dynamics in the COMFORT studies showed that the hemoglobin decreases that occurred with ruxolitinib treatment did not adversely affect the ruxolitinib-related benefit in OS; in addition, ruxolitinib treatment appeared to dilute the negative prognostic effect of lower baseline hemoglobin levels on survival.^26^

Overall, the rates of certain infections were higher in patients receiving ruxolitinib. A recent analysis has shown that ruxolitinib use may not predispose a patient to increased risk of infection, and patients who benefit in terms of spleen response may actually have a lower probability of developing an infection.^27^ However, because patients with MF are already predisposed to infections,^28^ additional precautions, including exclusion of patients with active infections, periodic screening for prevalent infections, and, where applicable, preventive measures such as antiviral prophylaxis, may be considered.

There was no pattern of SAEs after treatment discontinuation to suggest a withdrawal effect in this analysis. There was an apparent reduction in the risk of leukemic transformation with ruxolitinib; however, the different exposure times between treatment arms and subsequent treatments in the BAT group make this difficult to interpret. The relatively high rates of non-melanoma skin cancer observed here may be attributed to the extended duration of follow-up in the context of the high recurrence rates for these malignancies, especially considering the study population of patients who were previously treated with hydroxyurea. The causes of death are consistent with those reported in the overall population of patients with MF,^5, 29^ and there was no discernable pattern among the deaths that occurred in either treatment arm.

Consistent with the 2- and 3-year updates of COMFORT-II results,^14, 21^ ruxolitinib treatment prolonged OS compared with BAT. The reasons for the survival advantage observed with ruxolitinib cannot be addressed with these data but may be due to the number of benefits of treatment in terms of spleen size reduction,^25^ alleviation of cytokine-driven symptoms^8, 9^ and inflammation,^30^ improvement of overall nutritional status,^10^ and reduced fibrosis in some patients.^31^ Although several important baseline patient and clinical characteristics have been shown to be prognostic for worse survival in the currently used risk stratification systems (age >65 years, constitutional symptoms, hemoglobin <10 g/dl, white blood cell count >25 × 10^9^/l and circulating blasts ⩾1%)^5, 32^ and in a pooled analysis of the COMFORT data sets (baseline spleen volume, male sex, primary MF and lower platelet count),^25^ these characteristics appear not to have the same prognostic significance in the context of ruxolitinib treatment.^33^

Robust comparisons with BAT in an ITT analysis are difficult to make because the more recent benefits observed in the BAT arm were most likely driven by the large number of patients who were allowed to cross over and receive ruxolitinib. The more substantial reduction in the risk of death with ruxolitinib compared with BAT observed in the RPSFT analysis supports the assumption that this benefit is underestimated in the ITT analysis. Ideally, OS would be the primary end point in clinical trials as the gold standard for evaluating efficacy;^34^ however, this was not feasible at the time the COMFORT studies were initiated. Because survival in MF is measured in years rather than weeks, delaying an effective treatment for patients who experience a high symptom burden or depriving patients of the opportunity to cross over to a treatment that has proven benefit would have been considered unethical.

This final analysis of the first JAK1/JAK2 inhibitor approved in MF supports the safe and effective use of ruxolitinib in the long-term management of patients with MF.

## Figures and Tables

**Table 1:** Patient disposition at completion of the COMFORT-II study (5-year final analysis)

n *(%)*	*Ruxolitinib (*n=*146)*	*BAT (*n=*73)*	*Ruxolitinib after crossover from BAT (*n=*45)*
Completed 5 years of treatment/follow-up^a^	39 (26.7)	0	11 (24.4)
Discontinued	107 (73.3)	28 (38.4)	—
*Crossed over*^b^	—	45 (61.6)	—
After qualifying progression event	—	27 (37.0)	—
After protocol amendment 5	—	12 (16.4)	—
Other^c^	—	6 (8.2)	—
Discontinued after crossover	—	—	34 (75.6)
*Primary reasons for discontinuation*			
Adverse event	35 (24.0)	5 (6.8)	10 (22.2)
Consent withdrawn	10 (6.8)	9 (12.3)	0
Protocol deviation	2 (1.4)	0	5 (11.1)
Disease progression	32 (21.9)	4 (5.5)	7 (15.6)
Noncompliance with study medication	4 (2.7)	0	1 (2.2)
Noncompliance with study procedures	0	1 (1.4)	0
Unsatisfactory therapeutic effect	8 (5.5)	0	5 (11.1)
Other^d^	16 (11.0)	9 (12.3)	6 (13.3)

a After completing 5 years of treatment/follow-up on study, patients may have continued ruxolitinib treatment via commercially available product or enrollment in a compassionate use program.

b Patients randomized to the best available therapy (BAT) arm could cross over to receive ruxolitinib upon a protocol-defined progression event at any time during the study. Patients randomized to the ruxolitinib arm could continue receiving ruxolitinib in the extension phase upon a protocol-defined progression event at any time during the study if they were still deriving clinical benefit from ruxolitinib treatment, as assessed by the treating investigator. Qualifying progression events included progressive splenomegaly (defined per protocol as a ⩾ 25% increase in spleen volume from on-study nadir, including baseline) and the need for splenectomy. The study protocol was amended in January 2011 (amendment 5), after the primary analysis, to allow all patients to enter the extension phase and continue receiving ruxolitinib, including those who did not meet protocol-defined criteria for disease progression.

c Other reasons included six patients who crossed over from BAT to ruxolitinib without experiencing qualifying progression events before implementation of protocol amendment 5; of these patients, five discontinued due to protocol deviations and one discontinued due to other reason.

d Other reasons for discontinuation in the ruxolitinib arm included patients who underwent stem cell transplant (*n*=5; study days 131, 176, 239, 295 and 392), interruption of study medication for >8 weeks (*n*=2), lack of efficacy (*n*=2), meeting protocol-defined imaging discontinuation criteria (*n*=2), investigator decision (*n*=2), diagnosis of lung cancer with the start of chemotherapy treatment (*n*=1), unspecified safety event (*n*=1) and modest spleen response (*n*=1). Other reasons in the BAT arm included investigator decision (*n*=3), stem cell transplant (*n*=2; study days 34 and 168), patient decision (*n*=1), splenic irradiation (*n*=1), splenectomy (*n*=1) and thrombocytopenia as sign of disease progression (*n*=1). Other reasons for discontinuation after crossover included stem cell transplant (*n*=1; study days 1656 and 1159 after crossover), investigator decision (*n*=1), withdrawal of consent (*n*=1), unwillingness to undergo magnetic resonance imaging (*n*=1), initiating treatment with hydroxyurea (*n*=1) and enrollment in a ruxolitinib compassionate use program (*n*=1).

**Table 2:** Exposure-adjusted rates (per 100 patient-years) of common non-hematologic adverse events

*Preferred term,* n *(exposure-adjusted rate)*^a^^,^^b^	*Ruxolitinib randomized (*n*=146)*	*Ruxolitinib randomized+extension (*n*=146)*	*BAT randomized (*n*=73)*	*Ruxolitinib crossover (*n*=45)*	*Total ruxolitinib^c^ (*n*=191)*
Patient-years of exposure	170.12	409.52	66.98	79.70	489.22
Diarrhea	38 (22.3)	56 (13.7)	13 (19.4)	12 (15.1)	68 (13.9)
Peripheral edema	33 (19.4)	55 (13.4)	21 (31.4)	8 (10.0)	63 (12.9)
Dyspnea	24 (14.1)	37 (9.0)	15 (22.4)	12 (15.1)	49 (10.0)
Asthenia	28 (16.5)	38 (9.3)	9 (13.4)	10 (12.5)	48 (9.8)
Cough	22 (12.9)	38 (9.3)	12 (17.9)	10 (12.5)	48 (9.8)
Pyrexia	22 (12.9)	39 (9.5)	7 (10.5)	8 (10.0)	47 (9.6)
Bronchitis	18 (10.6)	41 (10.0)	6 (9.0)	3 (3.8)	44 (9.0)
Fatigue	23 (13.5)	36 (8.8)	8 (11.9)	8 (10.0)	44 (9.0)
Nasopharyngitis	27 (15.9)	40 (9.8)	9 (13.4)	4 (5.0)	44 (9.0)
Arthralgia	19 (11.2)	30 (7.3)	8 (11.9)	7 (8.8)	37 (7.6)
Nausea	21 (12.3)	30 (7.3)	7 (10.5)	5 (6.3)	35 (7.2)
Pain in extremity	18 (10.6)	24 (5.9)	4 (6.0)	11 (13.8)	35 (7.2)
Weight increase	23 (13.5)	29 (7.1)	1 (1.5)	5 (6.3)	34 (6.9)
Muscle spasms	15 (8.8)	28 (6.8)	5 (7.5)	4 (5.0)	32 (6.5)
Headache	18 (10.6)	23 (5.6)	4 (6.0)	8 (10.0)	31 (6.3)
Night sweats	14 (8.2)	27 (6.6)	6 (9.0)	4 (5.0)	31 (6.3)
Vomiting	16 (9.4)	27 (6.6)	1 (1.5)	4 (5.0)	31 (6.3)
Abdominal pain	17 (10.0)	26 (6.3)	13 (19.4)	4 (5.0)	30 (6.1)
Back pain	18 (10.6)	24 (5.9)	10 (14.9)	4 (5.0)	28 (5.7)
Dizziness	12 (7.1)	20 (4.9)	5 (7.5)	6 (7.5)	26 (5.3)
Hematoma	15 (8.8)	22 (5.4)	3 (4.5)	4 (5.0)	26 (5.3)
Urinary tract infection	11 (6.5)	19 (4.6)	2 (3.0)	7 (8.8)	26 (5.3)
Decreased appetite	6 (3.5)	20 (4.9)	4 (6.0)	4 (5.0)	24 (4.9)
Epistaxis	13 (7.6)	18 (4.4)	5 (7.5)	6 (7.5)	24 (4.9)
Abdominal pain upper	12 (7.1)	16 (3.9)	4 (6.0)	6 (7.5)	22 (4.5)
Hypertension	9 (5.3)	20 (4.9)	3 (4.5)	2 (2.5)	22 (4.5)
Constipation	12 (7.1)	19 (4.6)	4 (6.0)	2 (2.5)	21 (4.3)
Herpes zoster	9 (5.3)	16 (3.9)	0	5 (6.3)	21 (4.3)
Paresthesia	11 (6.5)	17 (4.2)	4 (6.0)	4 (5.0)	21 (4.3)
Pruritus	9 (5.3)	17 (4.2)	13 (19.4)	4 (5.0)	21 (4.3)
Gastroenteritis	11 (6.5)	17 (4.2)	1 (1.5)	1 (1.3)	18 (3.7)
Insomnia	9 (5.3)	13 (3.2)	7 (10.5)	5 (6.3)	18 (3.7)
Cystitis	9 (5.3)	15 (3.7)	3 (4.5)	1 (1.3)	16 (3.3)

a Adverse events occurring in ⩾10% of patients in any group, regardless of the relationship to study drug.

b Adjusted rates were calculated as the number of events per 100 patient-years of exposure.

c Includes all patients who received a dose of ruxolitinib on study, including during randomized treatment, in the extension phase, or after crossover from the best available therapy (BAT) arm.

**Table 3:** Exposure-adjusted rates (per 100 patient-years) of grade 3/4 adverse events

*Preferred term,* n *(exposure-adjusted rate)*^a^^,^^b^	*Ruxolitinib randomized (*n=*146)*	*Ruxolitinib randomized + extension (*n=*146)*	*BAT randomized (*n=*73)*	*Ruxolitinib crossover (*n=*45)*	*Total ruxolitinib*^c^ *(*n=*191)*
Any AE	71 (41.7)	104 (25.4)	24 (35.8)	26 (32.6)	130 (26.6)
Anemia	21 (12.3)	31 (7.6)	5 (7.5)	12 (15.1)	43 (8.8)
Thrombocytopenia	14 (8.2)	20 (4.9)	4 (6.0)	9 (11.3)	29 (5.9)
Pneumonia	2 (1.2)	10 (2.4)	4 (6.0)	1 (1.3)	11 (2.2)
General physical health deterioration	2 (1.2)	5 (1.2)	3 (4.5)	3 (3.8)	8 (1.6)
Acute renal failure	3 (1.8)	4 (1.0)	0	3 (3.8)	7 (1.4)^d^

Abbreviation: AE, adverse event.

a Adverse events occurring in ⩾5% of patients in any group, regardless of relationship to study drug.

b Adjusted rates were calculated as the number of events per 100 patient-years of exposure.

c Includes all patients who received a dose of ruxolitinib on study, including during randomized treatment, in the extension phase, or after crossover from the best available therapy (BAT) arm.

d No relationship between acute renal failure events and study drug was suspected by the investigators.
