# Supplementary material for: Long-term findings from COMFORT-II, a phase 3 study of ruxolitinib vs best available therapy for myelofibrosis
Source: Leukemia. 2016 Jun 17;30(8):1701–7. doi: 10.1038/leu.2016.148 (PMC5399157; doi:10.1038/leu.2016.148)
Supplement: Supplementary Information [file leu2016148x1.doc]

**Supplemental Material**

COMFORT-II Investigators

**Austria:** *Medizinische Universität, Wien—*H. Gisslinger; *Landeskrankenhaus Salzburg, Salzburg—*R. Greil; *Krankenhaus der Barmherzigen Schwestern Linz, Linz—*H. Rumpold; *Universitätsklinik Innsbruck, Innsbruck—*G. Gastl; **Belgium:** *Cliniques Universitaires Saint-Luc, UCL, Brussels—*L. Knoops; *Cliniques Universitaires UCL de Mont-Godinne, Yvoir—*A. Bosly; *ZNA Middelheim, Antwerp—*R. De Bock; *Centre Hospitalier de Jolimont-Lobbes, La Louvière—*A. Delannoy; *UZ Leuven/Campus Gasthuisberg, Leuven—*T. Devos; *AZ St-Jan AV, Brugge—*J. Van Droogenbroeck; *Oncologisch Centrum—AZ Groeningen, Kortrijk—*K. Van Eygen; *ZNA Stuivenberg, Antwerpen—*P. Zachee; *H Hartziekenhuis Roselare-Menen vzw, Roeselare—*H. Demuynck; *Yirga Jesseziekenhuis, Hasselt—*K. Theunissen; **France:** *Hôpital Saint Louis, Paris—*J.-J. Kiladjian; *Centre Hospitalo Universitaire de Grenoble, Grenoble—*J.-Y. Cahn; *Hôpital Claude Huriez, CHRU de Lille, Lille—*N. Cambier; *Hôpital Saint Antoine, Paris—*N. Cassadevall; *Centre Hospitalier de Lens, Lens—*B. Dupriez; *Hôpital Haut- Lévêque, Pessac—*N. Milpied; *CHU Carémeau Nimes, Nimes—*E. Jourdan; *CHU Côte de Nacre, Caen—*K. Benabed; *CHU Strasbourg,* *Hôpital Civil, Strasbourg—*S. Natarajan-Amé; *CHU Purpan, Toulouse—*C. Récher; *Institut Gustave Roussy, Villejuif—*V. Ribrag; *Institut Paoli-Calmettes, Marseille—*N. Vey; **Germany:** *Robert-Bosch-Krankenhaus, Stuttgart—*W. Aulitzky; *University Hospital Essen, University of Duisburg-Essen, Essen—*J. Novotny; *Universitätsmedizin Mainz, Main, Mainz—*T. Kindler; *Universitätsklinikum Münster, Münster—*T. Sauer; *Charité, Universitätsmedizin Berlin, Berlin—*P. Le Coutre; *Universitätsklinikum Leipzig AöR, Leipzig—*D. Niederwieser and H. K. Al-Ali (SI); *Johann-Wolfgang-Goethe Universität Frankfurt, Frankfurt—*O. Ottmann; *Klinikum der Universität zu Köln, Köln—*C. Scheid; *Universitätsklinikum Tübingen, Tübingen—*M. Sökler; *Zentrum Innere Medizin, Magdeburg—*F. Heidel; **Italy:** *Fondazione IRCCS Policlinico San Matteo, Pavia—*G. Barosi and F. Passamonti; *University of Milano, Bicocca San Gerardo Hospital, Monza—*C. Gambacorti-Passerini; *Az. Ospedaliera “S. Luigi Gonzaga,” Orbassano—*D. Cilloni; *University of Florence, Hospital Careggi, Florence—*A.M. Vannucchi and L. Pieri; *Hospital Niguarda Cà Granda, Milano—*E. Pungolino; **The** **Netherlands:** *University Medical Center St Radboud, Nijmegen—*N. Schaap; *Rijksuniversiteit Groningen, Groningen—*J.C. Kluin-Nelemans; *Maastricht Universitair Medisch Centrum, Maastricht—*H. Schouten; *Erasmus MC, Center Location, Rotterdam—*P. te Boekhorst; *HagaZiekenhuis, The Hague—*P. Wijermans; **Spain:** *Hospital Clinic, IDIBAPS, University of Barcelona, Barcelona—*F. Cervantes; *Hospital Clinico Universitario de Valencia, Valencia—*J.-C. Hernandez-Boluda; *Hospital Universitario Puerta de Hierro, Madrid—*E. Ojeda; **Sweden:** *Sahlgrenska University Hospital, Gothenburg—*P. Johansson; *Karolinska University Hospital, Huddinge, Stockholm*—D.Tesfa; **United Kingdom:** *Guy's Hospital, London—*C.N. Harrison; *Addenbrooke’s Hospital, Cambridge—*A. Green; *Belfast City Hospital, Belfast—*M. McMullin; *Oxford Radcliffe Hospitals NHS Trust* *(Churchill Hospital)*, *Oxford—*P. Vyas

**Supplemental Table 1. Shift table for fibrosis grade by treatment**

| **Last available postbaseline fibrosis grade** | **Ruxolitinib**  **(n = 146)** | | | | | **BAT^a^**  **(n = 73)** | | | | |
| --- | --- | --- | --- | --- | --- | --- | --- | --- | --- | --- |
|  | **Baseline fibrosis grade, n (%)** | | | | | **Baseline fibrosis grade, n (%)** | | | | |
|  | **0** | **1** | **2** | **3** | **Missing** | **0** | **1** | **2** | **3** | **Missing** |
| **0** | **1 (0.7)** | **1 (0.7)** | **2 (1.4)** | **1 (0.7)** | **2 (1.4)** | **0** | **0** | **0** | **0** | **0** |
| **1** | **0** | **10 (6.8)** | **9 (6.2)** | **2 (1.4)** | **0** | **0** | **1 (1.4)** | **0** | **1 (1.4)** | **0** |
| **2** | **0** | **2 (1.4)** | **8 (5.5)** | **8 (5.5)** | **1 (0.7)** | **0** | **0** | **4 (5.5)** | **1 (1.4)** | **0** |
| **3** | **0** | **6 (4.1)** | **19 (13.0)** | **28 (19.2)** | **2 (1.4)** | **0** | **0** | **4 (5.5)** | **8 (11.0)** | **3 (4.1)** |
| **Missing** | **2 (1.4)** | **2 (1.4)** | **17 (11.6)** | **20 (13.7)** | **3 (2.1)** | **2 (2.7)** | **2 (2.7)** | **19 (26.0)** | **24 (32.9)** | **4 (5.5)** |

^a^ Assessments after crossover from BAT to ruxolitinib are excluded.

**Supplemental Table 2. Causes of death occurring on treatment or within 28 days of treatment discontinuation**

| **Preferred term, n (%)^a^** | **Ruxolitinib randomized (n = 146)** | **Ruxolitinib randomized + extension (n = 146)** | **BAT randomized (n = 73)** | **Ruxolitinib crossover (n = 45)** | **Total ruxolitinib^b^ (n = 191)** |
| --- | --- | --- | --- | --- | --- |
| Any cause | 8 (5.5) | 17 (11.6) | 4 (5.5) | 4 (8.9) | 21 (11.0) |
| Arteriosclerosis coronary artery | 0 | 1 (0.7) | 0 | 0 | 1 (0.5) |
| Ascites | 0 | 0 | 0 | 1 (2.2) | 1 (0.5) |
| Cardiac arrest | 1 (0.7) | 1 (0.7) | 0 | 0 | 1 (0.5) |
| Cardiac failure | 1 (0.7) | 1 (0.7) | 1 (1.4) | 1 (2.2) | 2 (1.0) |
| Cardiopulmonary failure | 0 | 1 (0.7) | 0 | 0 | 1 (0.5) |
| Cerebral hemorrhage | 1 (0.7) | 1 (0.7) | 0 | 0 | 1 (0.5) |
| Cerebrovascular accident | 0 | 1 (0.7) | 0 | 0 | 1 (0.5) |
| Depressed level of consciousness | 0 | 1 (0.7) | 0 | 0 | 1 (0.5) |
| Disease progression | 1 (0.7) | 1 (0.7) | 0 | 0 | 1 (0.5) |
| Endocarditis | 1 (0.7) | 1 (0.7) | 0 | 0 | 1 (0.5) |
| Enteritis | 0 | 0 | 0 | 1 (2.2) | 1 (0.5) |
| Enterococcal sepsis | 0 | 1 (0.7) | 0 | 0 | 1 (0.5) |
| Febrile infection | 0 | 1 (0.7) | 0 | 0 | 1 (0.5) |
| Gastric cancer | 0 | 1 (0.7) | 0 | 0 | 1 (0.5) |
| Hepatic failure | 1 (0.7) | 1 (0.7) | 0 | 0 | 1 (0.5) |
| Intestinal perforation | 1 (0.7) | 1 (0.7) | 0 | 0 | 1 (0.5) |
| Klebsiella sepsis | 0 | 0 | 0 | 1 (2.2) | 1 (0.5) |
| Metastases to peritoneum | 0 | 1 (0.7) | 0 | 0 | 1 (0.5) |
| Multiorgan failure | 1 (0.7) | 1 (0.7) | 0 | 1 (2.2) | 2 (1.0) |
| Myocardial infarction | 0 | 1 (0.7) | 0 | 0 | 1 (0.5) |
| Performance status decreased | 0 | 1 (0.7) | 0 | 0 | 1 (0.5) |
| Pneumonia | 0 | 2 (1.4) | 0 | 0 | 2 (1.0) |
| Portal vein thrombosis | 1 (0.7) | 1 (0.7) | 0 | 0 | 1 (0.5) |
| Postoperative respiratory distress | 1 (0.7) | 1 (0.7) | 0 | 0 | 1 (0.5) |
| Pulmonary edema | 0 | 0 | 0 | 1 (2.2) | 1 (0.5) |
| Renal failure acute | 0 | 0 | 0 | 1 (2.2) | 1 (0.5) |
| Renal impairment | 0 | 0 | 1 (1.4) | 0 | 0 |
| Respiratory failure | 0 | 0 | 2 (2.7) | 0 | 0 |
| Respiratory failure, acute | 0 | 0 | 0 | 1 (2.2) | 1 (0.5) |
| Retroperitoneal hemorrhage | 1 (0.7) | 1 (0.7) | 0 | 0 | 1 (0.5) |
| Sepsis | 0 | 0 | 0 | 1 (2.2) | 1 (0.5) |
| Septic shock | 1 (0.7) | 1 (0.7) | 0 | 0 | 1 (0.5) |
| Skin infection | 0 | 1 (0.7) | 0 | 0 | 1 (0.5) |

^a^ A patient can have multiple reasons for death and can be included in more than 1 category.

^b^ Includes all patients who received a dose of ruxolitinib on study, including during randomized treatment, in the extension phase, or after crossover from the best available therapy (BAT) arm.

**Supplemental Figure 1. Patient disposition at completion of the COMFORT-II study (5-year final analysis)**


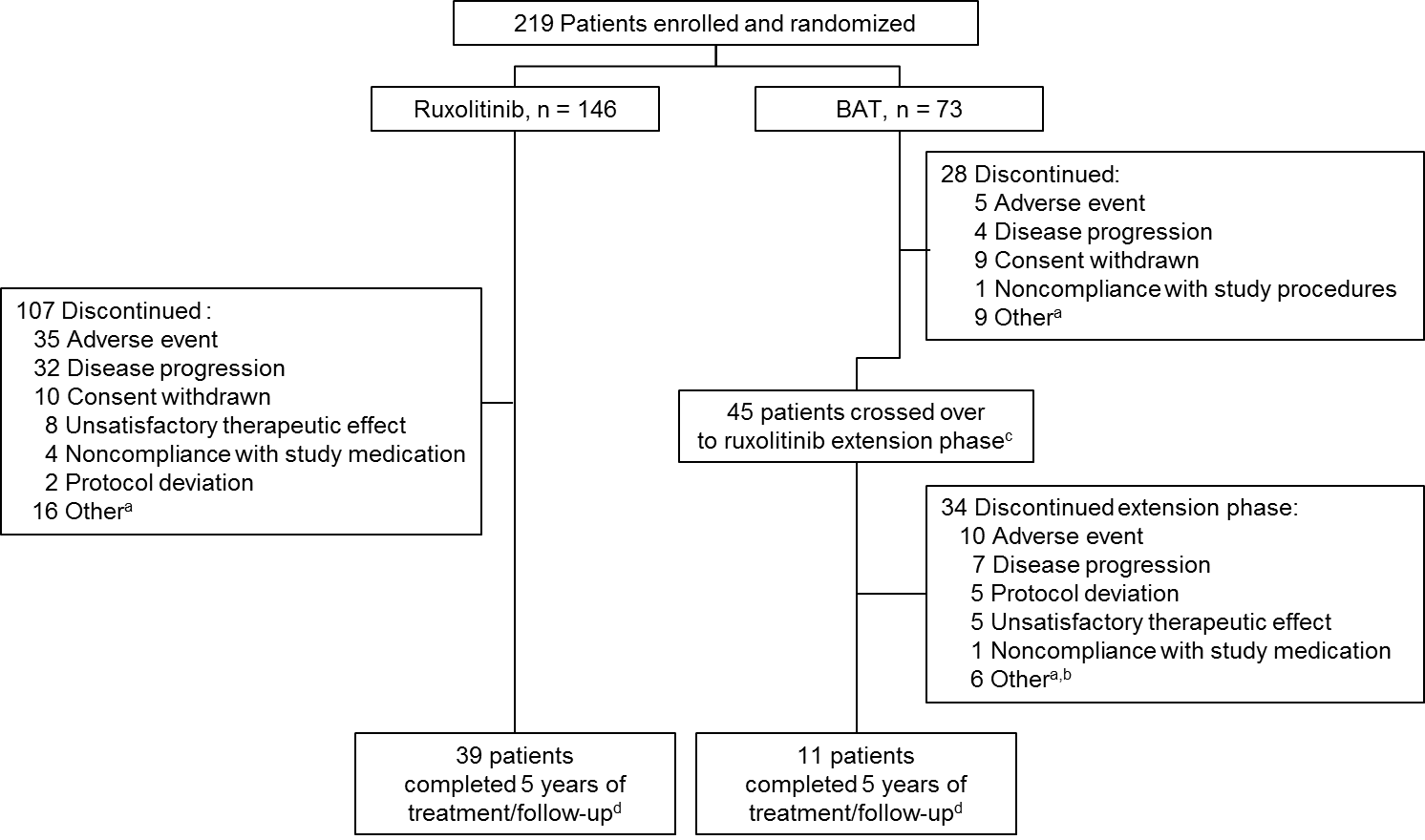


^a^ Other reasons for discontinuation in the ruxolitinib arm included stem cell transplant (n = 5), interruption of study medication for > 8 weeks (n = 2), lack of efficacy (n = 2), meeting protocol-defined imaging discontinuation criteria (n = 2), investigator decision (n = 2), diagnosis of lung cancer with the start of chemotherapy treatment (n = 1), unspecified safety event (n = 1), and modest spleen response (n = 1). Other reasons in the BAT arm included investigator decision (n = 3), stem cell transplant (n = 2), patient decision (n = 1), splenic irradiation (n = 1), splenectomy (n = 1), and thrombocytopenia as sign of disease progression (n = 1). Other reasons for discontinuation after crossover included stem cell transplant (n = 1), investigator decision (n = 1), withdrawal of consent (n = 1), unwillingness to undergo magnetic resonance imaging (n = 1), initiating treatment with hydroxyurea (n = 1), and enrollment in a ruxolitinib compassionate use program (n = 1).

^b^ Other reasons included 6 patients who crossed over from BAT to ruxolitinib without experiencing qualifying progression events before implementation of protocol amendment 5; of these patients, 5 discontinued due to protocol deviations and 1 discontinued due to other reason.

^c^ Patients randomized to the best available therapy (BAT) arm could cross over to receive ruxolitinib upon a protocol-defined progression event at any time during the study. Patients randomized to the ruxolitinib arm could continue receiving ruxolitinib in the extension phase upon a protocol-defined progression event at any time during the study if they were still deriving clinical benefit from ruxolitinib treatment, as assessed by the treating investigator. Qualifying progression events included progressive splenomegaly (defined per protocol as a ≥ 25% increase in spleen volume from on-study nadir, including baseline) and the need for splenectomy. The study protocol was amended in January 2011 (amendment 5), after the primary analysis, to allow all patients to enter the extension phase and continue receiving ruxolitinib, including those who did not meet protocol-defined criteria for disease progression.

^d^ After completing 5 years of treatment/follow-up on study, patients may have continued ruxolitinib treatment via commercially available product or enrollment in a compassionate use program.
